# Development and characterization of two human triple‐negative breast cancer cell lines with highly tumorigenic and metastatic capabilities

**DOI:** 10.1002/cam4.616

**Published:** 2016-01-18

**Authors:** Yanrong Su, Thomas J. Pogash, Theresa D. Nguyen, Jose Russo

**Affiliations:** ^1^ The Irma H. Russo, MD Breast Cancer Research LaboratoryFox Chase Cancer Center‐Temple University Health SystemPhiladelphiaPA19111USA

**Keywords:** triple‐negative breast cancer, tumorigenicity, epithelial–mesenchymal transition, cancer stem cell, EpCAM

## Abstract

Triple‐negative breast cancer (TNBC) is a group of cancer with high diversity, limited therapies, and poor prognosis. TNBC cell lines and animal models provide effective tools for studies and drug discovery. Here, we report the development of two TNBC cell lines (XtMCF and LmMCF) based on our existing cell model that consists of normal breast epithelial cell line MCF10F, estradiol‐transformed cells trMCF, and Boyden chamber‐selected tumorigenic cells bsMCF. The XtMCF and LmMCF cell line were derived from xenograft and lung metastasis of bsMCF cells, respectively. The bsMCF, XtMCF, and LmMCF cells have undergone epithelial–mesenchymal transition (EMT), exhibiting a mesenchymal‐like feature. In vivo studies showed XtMCF and LmMCF cells were highly tumorigenic and metastatic. The injection of 5 × 10^4^ cells to CB17/SCID mice mammary fat pad produced xenografts in 9/9 mice and tumors reached 10 millimeters in diameter in 5 weeks. The injection of 1 × 10^6^ XtMCF or 8 × 10^4^ LmMCF cells into the mice tail vein was sufficient to form extensive lung metastases in 4 weeks. The two new cell lines exhibited CD44^+^/CD49f^+^ and CD44^+^/EpCAM ^+^ cancer stem cell (CSC) characteristics, and the EGF‐like domain of EpCAM was cleaved off. Together with the normal and early transformed counterparts, herein we provide a complete cancer model for the study of initiation, evolution, and identification of new therapeutics for TNBC. The finding that EGF‐like domain of EpCAM was cleaved off in cells which have undergone EMT suggests this cleavage may be involved in the EMT process and the cancer stem cell properties of these cells.

## Introduction

Breast cancer is a malignant disease most frequently diagnosed in women. Triple‐negative breast cancer (TNBC), which tends to have a much poorer prognosis than other subtypes of breast cancer, accounts for 10–15% prevalence of cases. TNBC is a highly diverse group of cancers. These tumors are of higher histological grade, affect more young women, are more likely to recur early, and metastasize to distant sites 1. Treatment of patients with TNBC has been challenging due to the heterogeneity of the disease and absence of well‐defined molecular targets 2.

TNBC cell lines and related animal models are essential tools to develop therapeutics for TNBC. Of seventeen TNBC cell lines listed in American Type Culture Collection (ATCC) TNBC panel 3, seven cell lines including BT‐20, BT‐549, DU4475, HCC1806, MDA‐MB‐157, MDA‐MB‐231, and MDA‐MB‐468 are described to be tumorigenic in mice 3, 4, 5, 6, 7, 8. Besides these seven cell lines, two other TNBC cell lines, Sum149 and Sum159, are also widely used for in vivo studies 9, 10.

Compared to the diversity of TNBC, the number of available TNBC cell lines that can be used for in vivo studies is limited. In addition, these cell lines are usually established from the primary or metastatic tumors and lack parental cell lines at early stages. The transformation of normal cells to malignant cells is a multistep process that involves the accumulation of genetic and epigenetic changes 11. The use of a cell model in which normal cells are progressively transformed into malignant cells facilitates the identification and characterization of genes and pathways responsible for the progression thus providing new insights for the treatment. We have developed a unique cell model consisting of a series of cell lines and which presents with EMT during the progression 12, 13, 14. The baseline cell of this model is MCF10F, a spontaneously immortalized normal‐like triple‐negative human breast epithelial cell line 14. MCF10F cell line treated with 17‐*β* estradiol for two weeks exhibited features of transformation and was named trMCF. The trMCF cells were then plated in Boyden chambers, and the invaded cells were selected and named bsMCF. The bsMCF cell line showed characteristics of EMT; it was highly invasive in Matrigel chamber, and tumorigenic in SCID mice 13. bsMCF cells were also metastatic in SCID mice when injected into the tail vein. However, the development of lung metastases required injection of over 2 × 10^6^ cells/mouse which killed some mice during injection. Here, we report the development and characterization of two additional cell lines with high tumorigenic and metastatic capabilities. The two new cell lines, named as XtMCF and LmMCF, were derived from xenograft and lung metastasis of luciferase transfected bsMCF cells, respectively. Moreover, we demonstrated that XtMCF and LmMCF cells have undergone EMT and show CD44^+^/CD49f^+^ and CD44^+^/EpCAM^+^ CSC properties, and the EGF‐like domain of EpCAM in mesenchymal‐like cells is cleaved off. We also revealed that the Wnt signaling is activated during the progression of this cell model.

## Material and Methods

### Cell culture

MCF10F, trMCF, and bsMCF were maintained in DMEM:F12 supplemented media (Appendix S1). bsMCF cells were transfected with pGL4.51(luc2/CMV/Neo) vector (Promega, San Luis Obispo, CA) and maintained in media with 800 μg/mL G418 (so‐called bsMCF‐luc cells). MCF10F, T47D, MCF7, SK‐BR‐3, MDA‐MB‐231, MDA‐MB‐468, and Hs578t were from cell culture facility of FCCC. HCC1954 cell line was from American Type Culture Collection (ATCC). Sum149pt and Sum159pt were obtained from Asterand (Detroit, MI), and the media used for these cells are described in Appendix S1. All cell lines used for this study were used in less than ten passages after recovery.

### Deriving XtMCF and LmMCF cell lines

**Figure 1:**
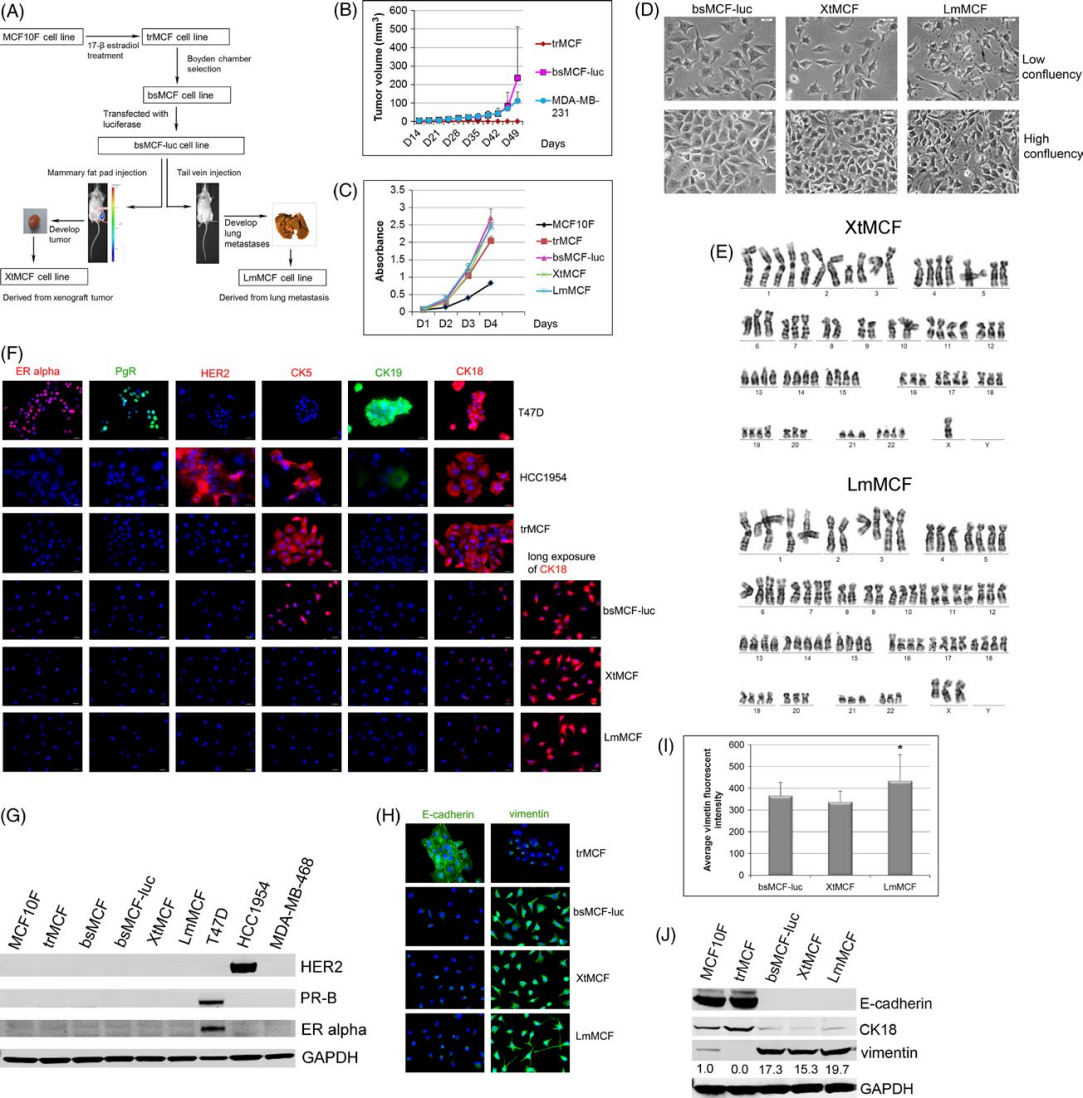
Development of two new TNBC cell lines. (A) Schematic representation of establishment of a TNBC model. (B) Tumor growth curves**.** CB17/SCID mice received a single injection of 3 × 10^6^ trMCF, bsMCF‐luc, or MDA‐MB‐231 cells to MFP. trMCF was not tumorigenic. bsMCF‐luc and MDA‐MB‐231 had similar tumor growth dynamic in first 6 weeks, and then, bsMCF‐luc exceeded MDA‐MB‐231. *X*‐axis: days after injection. (C) Cell growth curves by MTT assay. Cells were plated in 96‐well plate at 500 cells/well, and the proliferation was measured in four consecutive days starting from one day postplating. (D) Morphological images taken by phase contrast microscope. Arrow indicates the filopodium and enlarged head shown by LmMCF cells. Scale bar, 20 μm (200×). (E) Karyotype analysis of XtMCF and LmMCF cell line. One representative karyotype was shown from six karyotyped cells. (F) IF staining on cultured cells. The staining was overlapped with DAPI (blue) to show nuclei. CK18 fluorescence was exposed for 20 milliseconds for all cell lines and then 100 milliseconds (long exposure) to show the expression in bsMCF‐lus, XtMCF and LmMCF cells. Scale bar, 20 μm (400×). (G) WB analyses of ER alpha, PgR, and HER2. (H) IF staining of EMT markers. Scale bar, 20 μm (400×). bsMCF‐luc, XtMCF, and LmMCF underwent a complete EMT process. (I) Quantification of vimentin fluorescent staining in three mesenchymal‐like cells. The quantification was performed from 15 randomly selected fields. The experiment was repeated twice and one representative experiment was shown here. (I) WB analyses of EMT markers. The number below the band indicates the relative expression level to MCF10F.

To derive new cell lines, CB17/SCID mice at 50 days of age were used. Animals were purchased from the Laboratory Animal Facility at Fox Chase Cancer Center (FCCC) and maintained in the facility. Cells were injected using protocols approved by the Institutional Animal Care and Use Committee (IACUC) of FCCC. For the xenograft model, 3 × 10^6^ bsMCF‐luc cells were suspended in 1:1 mixture of PBS and Matrigel (BD Biosciences, San Jose, CA) to a volume of 0.2 mL and were injected into the mammary fat pad (MFP). Animals were palpated twice a week and sacrificed when tumors reached 10 millimeters (mm) in diameter. The xenograft was excised, cut into small pieces, and placed in cell culture media. The cell line derived from this culture was named XtMCF (Fig. 1A).

For the metastatic model, 2 × 10^6^ bsMCF‐luc cells suspended in 200 μL PBS were injected into tail vein. Mice were sacrificed eight weeks after cell injection. Tumor foci were carefully removed from the lungs, cut into small pieces, and placed in cell culture media. The cell line derived from this culture was named LmMCF (Fig. 1A).

Both XtMCF and LmMCF cell lines were maintained in the same medium used for parental cell line bsMCF‐luc. Cells were passaged every three days, karyotyped at passage 10, and used for studies after passage 10.

### MTT cell proliferation assay

Cell proliferation was assessed by measuring 3‐(4,5‐dimethylthiazol‐2‐Yl)‐2,5‐diphenyltetrazolium bromide (MTT) absorbance using Vybrant MTT Cell Proliferation Kit (Molecular Probes, Eugene, OR). Briefly, cells were plated in 96‐well plate at a density of 500 cells/well; the proliferation was measured in four consecutive days starting from one day postplating. Data were analyzed using SigmaPlot v12.

### Karyotyping

Karyotype analysis was carried out by Genetic Research Facility at FCCC. Twenty metaphases were counted and six cells were karyotyped for each cell line.

### Immunofluorescence (IF) staining

Cells were plated on chamber slides. After 3 days of culture, cells were fixed with 10% buffered formalin, permeabilized, and stained with antibodies to E‐cadherin, vimentin, ER alpha, PgR, HER2, CK5, CK18, CK19, CD24, CD44, EpCAM{Abbiotec}, EpCAM(VU1D9), EpCAM(E144), and beta‐catenin (Table S1). The details of staining are described in Appendix S1.

### Western blotting (WB)

Total cell lysates were prepared using radioimmunoprecipitation assay buffer (Cell Signaling Technology, Danvers, MA). Forty micrograms proteins was separated on 4–12% Bis‐Tris gels (Life Technologies, Grand Island, NY) and transferred to nitrocellulose membrane. E‐cadherin, vimentin, EpCAM(VU1D9), EpCAM(E144), CK18, and Tcf4 (Table S1) were detected using Li‐Cor Odyssey imaging system (Li‐Cor Biotechnologies Corporation, Lincoln, NE). GAPDH was used as loading control.

### Wound healing assay

Single‐cell suspensions were plated in 24‐well plate in four replicates at density of 1 × 10^5^ cells/well and incubated overnight to allow cells to reach confluence. The cell monolayer was scratched with a 200 μL GeneMate pipette tip (BioExpress, Kaysville, UT) to make the wound. Images were acquired using MetaMorph, Olympus IX‐71 microscope with automated stage at time point of 0 and 17 h. Three images were acquired from each well at each time point. The wound area was measured using MetaMorph (Molecular Devices, Sunnyvale CA). Data are presented as percent wound closed calculated using the following formula: percent wound closed = {(wound area at 0 h ‐ wound area at end time point)/wound area at 0 h} × 100.

### Ducts/solid masses formation in collagen

As previously described 13, cells were suspended in bovine type I collagen solution (PureCol; Advanced BioMatrix, San Diego, CA) and plated at 1500 cells/well in four replicates onto 24‐well plate precoated with collagen. The plate was put in 37°C incubator supplied with 5% CO2 to allow the solidification of collagen, and then, the culture media was added to each well. Cells were fed with fresh normal media every two days. The formation of the ducts or masses was examined under an inverted microscope. At the end of examination period, images were acquired using NIKON ECLIPSE TS100 microscope.

### Colony forming efficiency in methylcellulose

Colony formation assay was performed as previously described 15. Briefly, single cells were suspended in 0.8% methylcellulose dissolved in DMEM/F12 media and plated at 1500 cells/well in four replicates. At the end of assay, images were acquired using NIKON ECLIPSE TS100 microscope. The number and size of colonies were measured using MetaMorph. Detailed information is provided in the Appendix S1.

### In vivo tumorigenic and metastatic study

All animal studies were carried out using protocol approved by the IACUC of FCCC. Female CB17/SCID mice which were 8–9 weeks old were obtained from FCCC animal facility. For the tumorigenic study, a viable single‐cell suspension in 100 μL of PBS was mixed with 100 μL Matrigel and injected into the mouse MFP. Tumor masses were measured twice a week with a caliper. Tumor volume was calculated as follows: 0.5 × L × W^2^, where L and W are the large and smaller diameters. Mice were sacrificed when the tumor reached 10 mm in diameter. Tumors, livers, lungs, and brains were fixed in 10% neutral buffered formalin and processed for histological examination.

The lung‐metastasis model was established by injecting single‐cell suspension of XtMCF or LmMCF cells (in 200 µl of PBS) into the mouse tail vein. Mice were monitored three times a week and sacrificed when mice showed signs of distress. The lungs, livers, and brains were fixed in Bouin's solution. Whole mount images were acquired using Olympus camera with a stereo microscope.

### Immunohistochemical (IHC) staining

Tumor tissue microarray (TMA) was constructed using paraffin‐embedded xenografts and lung tissues. Human breast cancer tissues known to be ER, PgR, or HER2 positive were added to the same TMA block for validation of the staining. The use of human breast cancer samples received approval from the Institutional Review Board (IRB) of FCCC. Staining was performed following the standard protocol using i6000 Autostainer (BioGenex, Fremont, CA). Primary antibodies used are shown in Table S1. Super Sensitive^TM^ Polymer‐HRP Detection System (BioGenex, Fremont, CA) was used to detect the immunostaining.

### Tumorsphere formation assay

Single‐cell suspension was plated in ultralow‐attachment 6‐well plate at a density of 50,000 cells/well in culture media supplemented with 10 ng/mL EGF, 20 ng/mL bFGF, and 1 × B27. Four days after culture, images of tumorspheres were acquired using NIKON ECLIPSE TS100 microscope. The number of spheres was counted and graphed.

### Flow cytometric analysis

The following antibodies were used for flow cytometric analysis: anti‐CD44‐PE/Cy7 (Abcam); anti‐CD49f‐PE (clone GoH3); and anti‐CD133/2‐VioBright FITC (clone 293C3) (Miltenyi Biotec Inc, San Diego, CA). Cells were trypsinized, resuspended in 5% FBS/PBS/2 nm EDTA buffer, aliquoted to 2 × 10^6^ cells/100 μL per sample, incubated with Human Fc Receptor Binding Inhibitor Purified for 20 min, and then stained with indicated antibody for 30 min. OneComp eBeads stained with three individual antibodies were used to set up compensation. Samples were analyzed with BD FACSAria II (Becton‐Dickinson, Franklin Lakes, New Jersey), and 500,000 cells were captured from each sample. Data were analyzed with FlowJo software (BD).

### Statistical analysis

Statistical analysis was carried out with SigmaPlot 12.0 software (Systat Software Inc, San Jose, CA). Studies involving more than two groups were analyzed by one‐way analysis of variance (anova). Chi‐square analysis was used to evaluate the distribution of colonies for colony formation assay.

## Results

### Development of XtMCF and LmMCF cell lines

Our laboratory has established a cell model consisting of cell line MCF10F, trMCF, and bsMCF 12, 13, which represents the initiation and transformation of TNBC. The bsMCF cell line has undergone EMT completely; it is tumorigenic and metastatic in CB17/SCID mice. Due to the large number of cells per injection required to develop lung metastases which is inconvenient for in vivo studies, we sought to develop new derivative cell lines with higher tumorigenic and metastatic capacity. We established xenograft and metastatic model by injecting bsMCF‐luc cells into MFP or tail vein of female CB17/SCID mice. For xenograft model, all five mice injected with 3 × 10^6^ bsMCF‐luc cells developed xenografts (Fig. 1A,B). Xenografts from two mice were used to derive cell lines. Two cell lines were developed with no difference in cell morphology and expression of E‐cadherin and vimentin; thus, one of two cell lines was chosen to use in the following study and referred to as XtMCF cell line. For lung metastatic model, all five mice injected with 2 × 10^6^ bsMCF‐luc cells developed lung metastases. Two lung tumors from two individual mice were used to develop cell lines. Only one cell line was established and was named LmMCF cell line (Fig. 1A).

MTT assay showed trMCF, bsMCF‐luc, XtMCF, and LmMCF cell lines had roughly similar growth speeds (Fig. 1C). Morphologically, trMCF cells grew as interconnected colonies of polygonal cells. bsMCF cells were polygonal cells which grew as fibroblast‐like cells. There was no difference in morphology between bsMCF and bsMCF‐luc cells. XtMCF cells were very similar to bsMCF‐luc cells. LmMCF cell size was smaller than bsMCF‐luc, showing multiple elongated filopodia (usually more than four filopodia) and enlarged spin head at the tip of filopodia (Fig. 1D).

To check the chromosomal abnormalities, XtMCF and LmMCF cells at passage 10 were karyotyped. Both cell lines were aneuploidy female (Fig. 1E). For XtMCF, modal number was 76 to 80 (4n), range was 71 to 95. The karyotype was presented as: 76–80: XX,‐X,‐X,add(1)(p36.2),add(2)(q21?),‐2,der(3)t(3;?)(q11;?),del(3) (p13),‐7,‐8,‐8,‐9,‐9,+11,‐16,‐18,‐20,‐21{cp20}. For LmMCF, modal number was 79 to 83 (4n), and range was 68 to 83. The karyotype was presented as: 79–83: XX, ‐X,del(X)(q26),add(1)(p36.2)x2,‐2,der(3)t(3;?)(q11;?),der(9)t(1;9)(p11;q34),‐12,+14,‐18,‐20,‐21,‐22,der(22)t(1;22)(q10;p11){cp20}. Both of XtMCF and LmMCF had the addition of chromosome 1p36.2, which was one of the characteristic changes in MCF10F cell line, and was present in xenografts of bsMCF cells and cell lines derived from these xenografts 13.

### Molecular characterization of XtMCF and LmMCF cells

The two new cell lines were characterized using antibodies for the classification of breast cancer. As shown in Figure 1, trMCF, bsMCF‐luc, XtMCF, and LmMCF cells were negative for ER alpha, PgR, and HER2, indicating they are triple‐negative cells. CK5 was positive in 100% of trMCF cells and decreased by 54.3%±4.1% in bsMCF‐luc cells. XtMCF and LmMCF were negative for CK5. CK19 was negative in all four cell lines. CK18 was significantly reduced in bsMCF‐luc, XtMCF, and LmMCF cells compared to trMCF cells. The expression of ER alpha, PgR, and HER2 was confirmed by WB (Fig. 1G). The assessment of EMT status showed E‐cadherin was positive in trMCF cells but undetectable by IF staining in bsMCF‐luc, XtMCF, and LmMCF cells. Vimentin was positive only in 10.1%±1.3% of trMCF cells; in contrast, it was present in all bsMCF‐luc, XtMCF, and LmMCF cells (Fig. 1H). Quantification of fluorescent intensity of vimentin staining showed LmMCF cells have an increased vimentin expression compared to bsMCF‐luc and XtMCF cells (Fig. 1I). The expression of CK18, E‐cadherin, and vimentin was confirmed by WB (Fig. 1J). Based on these results, bsMCF‐luc, XtMCF, and LmMCF cell lines were classified as basal B TNBC cell lines 16.

### XtMCF and LmMCF cells differ from bsMCF‐luc cells in migration, growth behavior in collagen, and colony formation capacity

**Figure 2:**
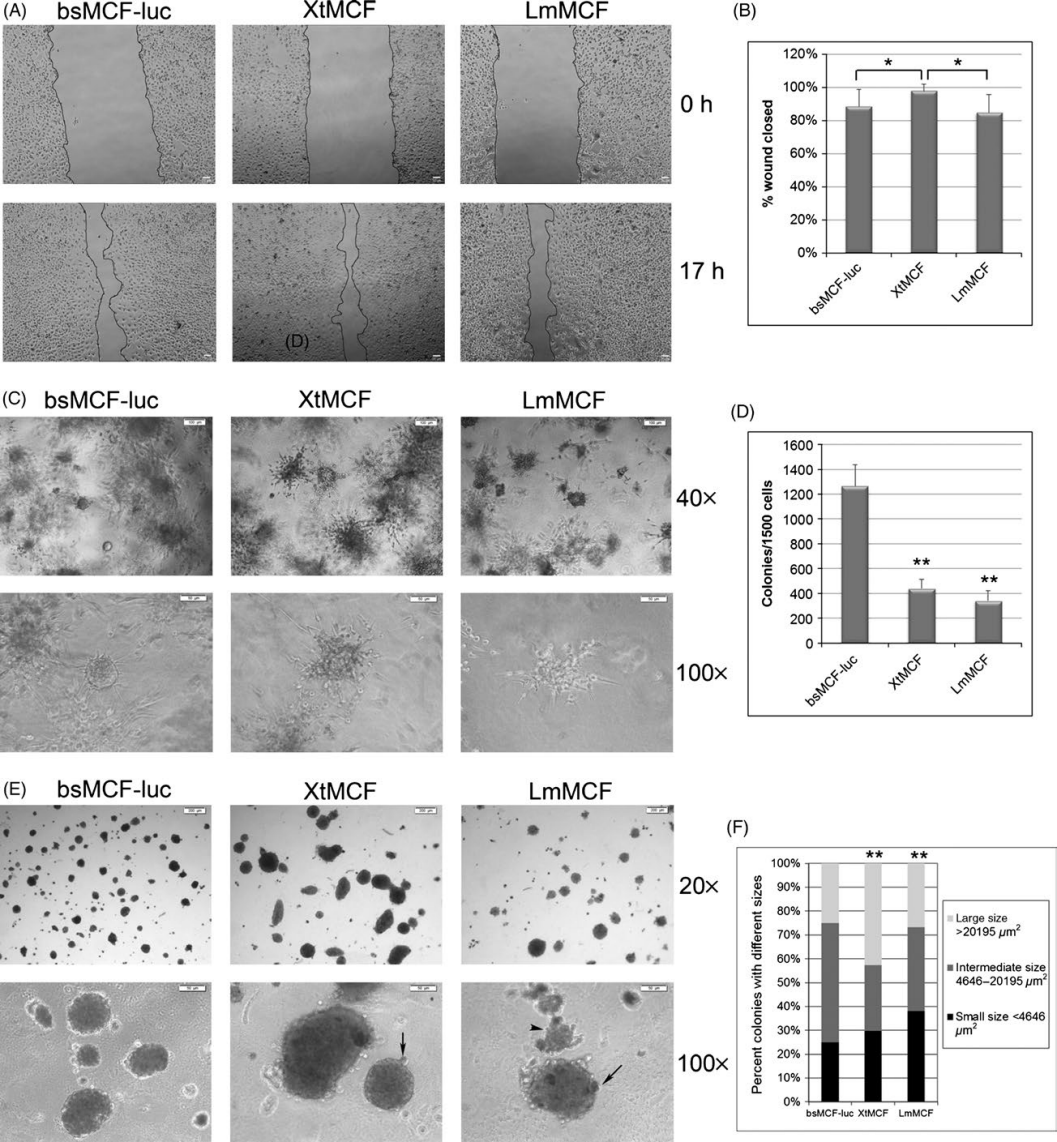
In vitro phenotypes of XtMCF and LmMCF cells. (A) Wound healing assay over 17 h of culture. Scale bar, 20 μm (100×). (B) Quantification of wound healing assay shows that XtMCF cells migrate faster than bsMCF‐luc and LmMCF cells. *indicates *P* < 0.05. (C) 3D culture in bovine type I collagen. Cells were mixed with collagen and plated onto precoated 24‐well plate at 1500 cells/well; pictures of structures formed in collagen were acquired after 6 days of culture. (D) XtMCF and LmMCF cells form fewer colonies than bsMCF‐luc cells in methylcellulose. **indicates *P* < 0.01 compared to bsMCF‐luc cells. Scale bar represents 100 μm for 40×, and 50 μm for 100× magnification. (E) Anchorage‐independent growth in methylcellulose. Single‐cell suspension was mixed with methylcellulose and plated onto agar coated 24‐well plate at 1500 cells/well. Pictures were taken after 10 days of culture. Scale bar represents 200 μm for 20×, and 50 μm for 100× magnification. (F) XtMCF cells form larger colonies than bsMCF‐luc and LmMCF, whereas LmMCF cells form smaller colonies than other cell lines in methylcellulose. **indicates *P* < 0.01 compared to bsMCF‐luc cells. All above experiments were repeated at least twice and one representative experiment was shown here.

We next examined whether XtMCF and LmMCF showed different phenotypes from bsMCF‐luc in vitro. Cell migration was investigated using wound healing assay (Fig. 2A). Quantification of cell movement over 17 h showed XtMCF cells migrated faster than bsMCF‐luc and LmMCF cells. There was no difference in migration between bsMCF‐luc and LmMCF cells (Fig. 2B).

We previously reported that MCF10F cells can form ducts in collagen which indicated these cells are well differentiated 17. bsMCF loses the ability to form ducts, but instead forms solid mass and grows in clusters in the collagen 15, 18. No difference was observed for the growth pattern of bsMCF and bsMCF‐luc cells in collagen. The masses formed by these two cell lines were usually tightly packed with protrusions invading into the surrounding collagen reminiscing the growth and invasion of primary tumors in vivo. Strikingly, XtMCF and LmMCF grew in clusters only; the formation of mass was very rare (Fig. 2C).

Anchorage‐independent growth of cells in agar is usually associated with tumorigenic potential. Our results showed that XtMCF and LmMCF cells formed significantly less number of colonies compared to bsMCF‐luc (Fig. 2D,E). The morphology of the colonies also varied among the three cell lines. Colonies of bsMCF‐luc and LmMCF cells were more circular, whereas about half of colonies of XtMCF cells were oval‐like (Fig. 2E). Notably, budding from the surface of colonies (arrows in Fig. 2E) was frequently observed in colonies from XtMCF and LmMCF, but not bsMCF‐luc cells. Besides that, LmMCF cells also grew as cell clusters or aggregates (arrow head in Fig. 2E) in methylcellulose. Quantification showed XtMCF formed larger colonies, whereas LmMCF formed smaller colonies compared to bsMCF‐luc cells (Fig. 2F).

### XtMCF and LmMCF cells are highly tumorigenic and metastatic in vivo

**Figure 3:**
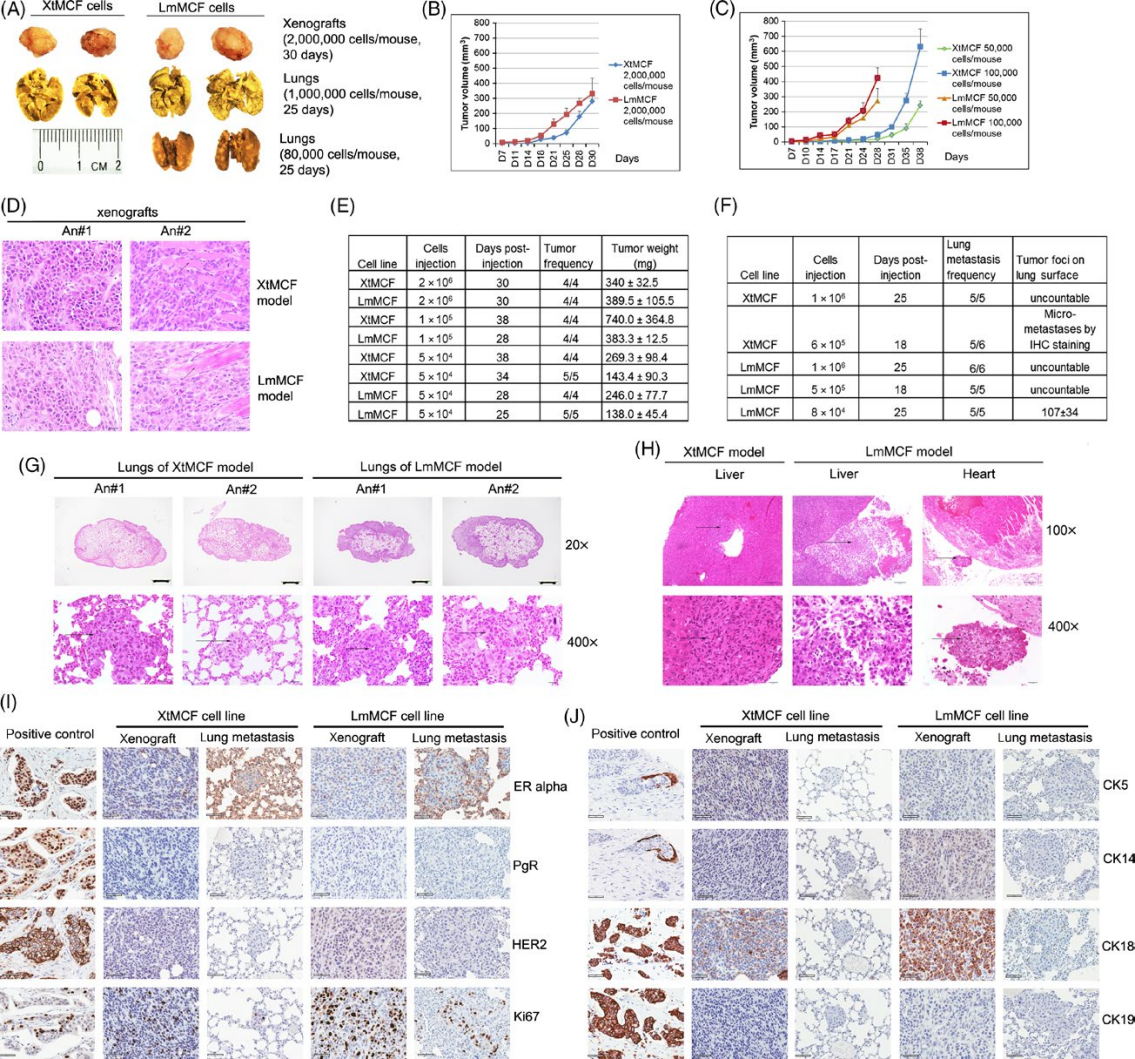
XtMCF and LmMCF cells display high tumorigenic and metastatic potential. (A) Representative pictures of xenografts and lungs fixed with Bouin's solution. Magnification, 6.3× for xenografts, 8× for lungs, scale bar was for the images of lungs. (B) Tumor growth curves. CB17/SCID mice received a single injection of 2 × 10^6^ cells to MFP; tumor growth was monitored twice a week. *X*‐axis: days after injection. (C) Tumor growth curves. CB17/SCID mice received a single injection of 1 × 10^5^ or 5 × 10^4^ cells to MFP; (D) H&E staining of xenografts, arrows indicate the muscle cells. Scale bar, 20 μm (400×). XtMCF and LmMCF tumor cells are highly invasive. (E) Evaluation of tumorigenicity. Cell number, days postinjection at sacrifice, tumor frequency (tumor formed/injected mice), and tumor weight at sacrifice are shown in table. (F) Evaluation of metastatic potential. Cell number, days postinjection at sacrifice, lung‐metastasis frequency (number of mice with lung metastasis/injected mice), and tumor foci on lung surface at sacrifice are shown in table. (G) H&E staining of lungs from the injection of 1 × 10^6^ cells into tail vein. LmMCF cells are more metastatic than XtMCF cells. Arrows indicate the metastases. Magnifications are shown in figure, and scale bar represents 10 mm for 20× magnification and 20 μm for 400× magnification. (H) H&E staining of liver and heart from the mice injected with tumor cells into tail vein. Arrows indicate metastases. Scale bar represents 100 μm for 100× magnification, represents 20 μm for 400× magnification. (I, J) The expression of ER, PgR, HER2, Ki67, CK5, CK14, CK18, and CK19 by IHC analyses in xenografts and lung metastases. Scale bar, 50 μm (400×). The results show tumor cells of XtMCF and LmMCF xenografts and lung metastases are TNBC cells with high proliferation rate.

Compared to xenografts of bsMCF‐luc and MDA‐MB‐231 cell lines (Fig. 1B), XtMCF and LmMCF xenografts grew significantly faster even with the injection of 2 × 10^6^ cells. The tumors started to grow exponentially three weeks postinjection and reached 10 mm in diameter in 30 days (Fig. 3A,B). These tumors were highly invasive; half of the tumors invaded to the skin or muscles of abdominal wall. Histological examination revealed they were poorly differentiated tumors and frequently invaded to muscles (Fig. 3D). To further evaluate the tumorigenic potential of these two cell lines, 1 × 10^5^ and 5 × 10^4^ cells were injected into MFP, and 100% mice formed tumors. The tumor growth of XtMCF was slower for the injection of 1 × 10^5^ and 5 × 10^4^ cells compared to the tumor growth when 2 × 10^6^ cells were injected. However, tumor growth of LmMCF cells was almost the same for all three cell concentrations (Fig. 3B,C). Tumor weights at sacrifice are shown in Figure 3E.

The metastatic capacity of the developed cells was evaluated by tail vein injection. With the injection of 1 × 10^6^ cells, the whole lung surface was filled with tumors and it was difficult to count tumor foci at sacrifice 25 days postinjection (Fig. 3A). The left lobe was more affected than the right lobes in all mice. Histological examination showed metastases present both on the lung surface and inside of the lung (Fig. 3F,G). The amount of metastasis foci was higher in the LmMCF model than in XtMCF model. Besides the metastases in the lung, 1/5 of mice in XtMCF model and 1/6 of mice in LmMCF model revealed metastases in the liver (Fig. 3H). Worth mentioning is the fact that 1/6 of mice in LmMCF model also showed metastasis to pericardium (Fig. 3H), one of the common sites for breast cancer metastasis 19. Strikingly, only 6 × 10^5^ XtMCF cells were able to form micrometastasis in the lungs of 5/6 mice 18 days postinjection. Remarkably, the lung surfaces of mice injected with 5 × 10^5^ LmMCF cells were filled with tumors even just 18 days postinjection. We then injected mice with 8 × 10^4^ LmMCF cells and sacrificed mice 25 days postinjection. The results showed that even this reduced number of cells was sufficient to form lung metastases in 100% (5/5) of mice (Fig. 3A,F).

### Classification of xenografts and lung metastases formed by XtMCF and LmMCF cells

IHC staining was performed to classify the xenografts and lung metastases. Consistent with the in vitro data (Fig. 1F), these xenografts and lung metastases were TNBC. The tumor cells were highly proliferative, and the rate of Ki67‐positive cells was 34.6 ± 9.1% and 40 ± 12.3% for XtMCF and LmMCF xenografts, respectively, and 21.6 ± 4.7% for LmMCF lung metastases (Fig. 3I). CK5, CK14, and CK19 were negative, suggesting tumors from XtMCF and LmMCF cell lines were basal‐like TNBC. Interestingly, CK18 was only positive in the xenografts but not present in lung metastases (Fig. 3J).

### XtMCF and LmMCF cells present CD44^+^/EpCAM^+^ cancer stem cell properties; EGF‐like domain of EpCAM is cleaved off

**Figure 4:**
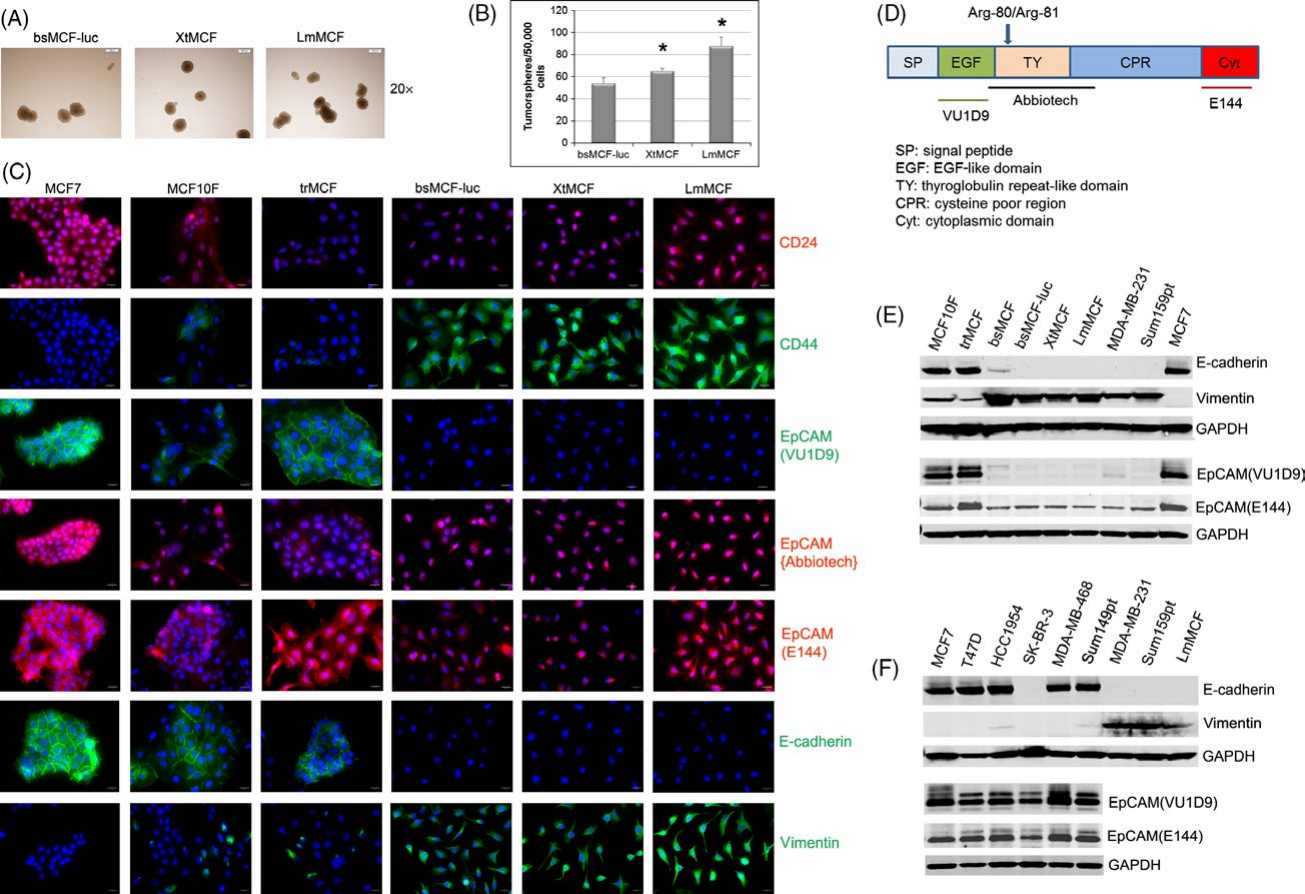
XtMCF and LmMCF cells present CD44^+^/EpCAM ^+^ cancer stem cell properties. (A) XtMCF and LmMCF are capable of forming tumorspheres cultured under nonadherent condition. Representative images of tumorspheres are shown. Scale bar: 200 μm (20×). (B) XtMCF and LmMCF produce more tumorspheres than parental bsMCF‐luc cells. LmMCF produces more tumorspheres than XtMCF cells. *P < 0.05 when compare to bsMCF‐luc, or when compare LmMCF to XtMCF. The experiment was repeated twice, one representative experiment was shown. (C) bsMCF‐luc, XtMCF, and LmMCF cells have undergone EMT process; they are CD44^+^/EpCAM+. The EpCAM in these cells does not have EGF‐like domain. Scale bar: 20 μm (400×). (D) EpCAM molecule and antibody map. The locations of epitopes of antibody VU1D9, Abbiotec, and E144 are indicated. Arrow: N‐terminal cleavage site between Arg‐80/Arg‐81. (E) bsMCF‐luc, XtMCF, LmMCF, MDA‐MB‐231, and Sum159pt cells have undergone EMT, do not have full length EpCAM expression examined by WB. (F) Full length EpCAM expression is observed only in epithelial breast cancer cell lines but not mesenchymal‐like cell lines by WB.

As XtMCF and LmMCF cells are highly tumorigenic and metastatic, we hypothesized these two cell lines are enriched for cancer stem cells (CSC). We performed tumorsphere formation assay, all three cell lines generated tumorspheres, demonstrating their stem‐like properties. Consistent with in vivo tumorigenicity data, both XtMCF and LmMCF cells produced more tumorspheres than bsMCF‐luc cells. Of note, LmMCF cells formed more spheres than XtMCF (Fig. 4A,B).

**Figure 5:**
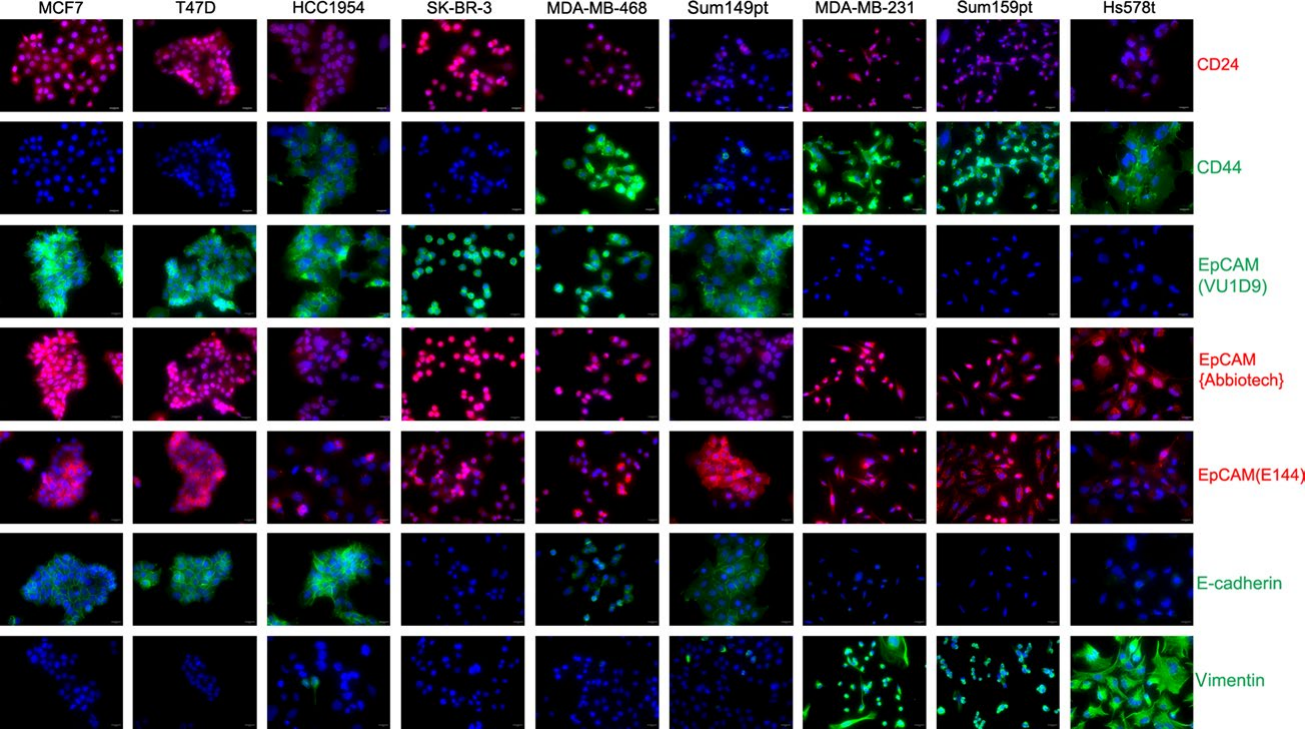
Expression of CD24, CD44, EpCAM, and EMT markers in breast cancer cell lines by IF staining. Cells were cultured on glass chamber slides and stained with indicated antibodies. The staining was overlapped with DAPI (blue) to show nuclei. MDA‐MB‐231, Sum159pt, and Hs578t cells have undergone EMT; they are CD44^+^/EpCAM ^+^. The EpCAM in these cells does not have EGF‐like domain. Scale bar: 20 μm (400×).

We next evaluated CSC markers CD24 and CD44 by IF staining. CD24 was weakly expressed in MCF10F, trMCF, bsMCF‐luc, and XtMCF cells; by contrast, it was moderately expressed in LmMCF cells. CD44 was weakly positive in some of MCF10F and trMCF cells. However, it was positive in all bsMCF‐luc, XtMCF, and LmMCF cells (Fig. 4C), confirming that EMT contributes to generate CD44^+^ cells 20. Quantification of CD44 fluorescent intensity did not show significant difference among bsMCF‐luc, XtMCF, and LmMCF cells. Next, we examined the expression of epithelial cell adhesion molecule (EpCAM). Initially, the EpCAM antibody we used was from Abbiotec. This antibody (referred to as EpCAM{Abbiotec}) recognizes amino acid (AA) 55 to 150 which corresponds to thyroglobulin repeat‐like domain and a part of cysteine poor region (Fig. 4D). IF staining showed that the staining mainly located in cytoplasm and nuclei, was weak in MCF10F and trMCF cells, whereas it was stronger in bsMCF‐luc, XtMCF, and LmMCF cells (Fig. 4C). This kind of staining pattern was observed in the study using the antibody to the thyroglobulin repeat‐like domain or cysteine poor region 21. As this antibody was not suitable for WB, the antibody EpCAM(VU1D9) which recognizes the EGF‐like domain at N‐terminal EpCAM was used. A strong 38 KDa band was detected in epithelial cell lines MCF7, trMCF, and MCF10F, and a weak band in MDA‐MB‐231. It was almost undetectable in mesenchymal‐like cell lines bsMCF‐luc, XtMCF, LmMCF, and Sum159pt (Fig. 4E). IF staining of EpCAM(VU1D9) revealed the expression was only shown in epithelial cells but not in mesenchymal‐like cells (Fig. 4C). To confirm the expression of EpCAM in mesenchymal‐like cells, the antibody EpCAM(E144), which recognizes the cytoplasmic tail of EpCAM molecule, was used. Immunoblotting showed EpCAM was expressed in all cells, but present lower level in mesenchymal‐like cells (Fig. 4E). The same result was seen by immunofluorescence using EpCAM(E144) (Fig. 4C). We next validated our observations in more breast cancer cell lines. Consistently, EpCAM(VU1D9) was not detected in mesenchymal‐like cell lines defined by CD24, CD44, E‐cadherin, and vimentin staining (Figs 4F and 5), and EpCAM{Abbiotec} and EpCAM(E144) were detected in all cell lines, shown stronger expression in luminal cell lines. These data suggest that EGF‐like domain at N‐terminal EpCAM is cleaved off in cells which have undergone EMT.

**Figure 6:**
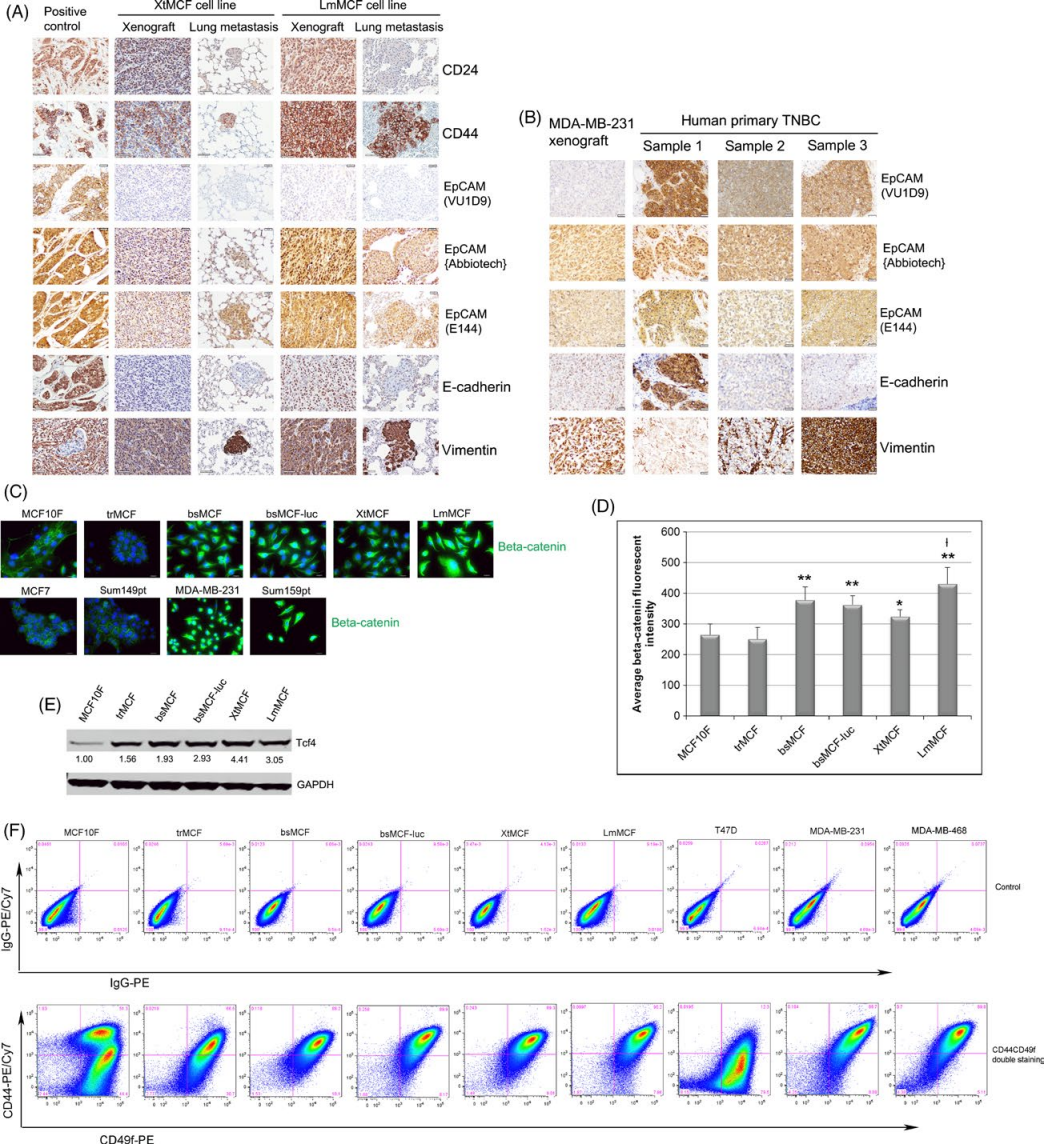
Confirmation of EpCAM expression in xenografts and lung metastases as well as human TNBC samples; Detection of Wnt signaling and CD44/CD49 double‐positive cells in the cell model. (A, B) TMA sections were stained with CD24, CD44, EpCAM, and EMT markers by IHC. Tumor cells of XtMCF, LmMCF, and MDA‐MB‐231 xenografts or lung metastases have undergone EMT, the EGF‐like domain of EpCAM is cleaved off in these cells. Human primary TNBC tumor cells that lost E‐cadherin or underwent EMT show reduced EpCAM expression. Scale bar represents 50 μm (400×) for CD24, CD44, E‐cadherin and vimentin in panel A, and represents 20 μm (200×) for EpCAM in panel A, and all images in panel B. (C) IF staining of beta‐catenin in cultured cells. Beta‐catenin is located on cell membrane of epithelial cells whereas translocate to cytoplasm and nuclei in mesenchymal‐like cells. Scale bar, 20 μm (200×). (D)Quantification of beta‐catenin fluorescent intensity measured with MetaMorph. *indicates *P* < 0.05 when compared to MCF10F; **indicates *P* < 0.01 when compared to MCF10F; ł indicates *P* < 0.01 when compared LmMCF to bsMCF‐luc and XtMCF. The quantification was performed from 15 randomly selected fields. The experiment was repeated twice and one representative experiment was shown here. (D) WB shows Tcf4 is elevated in transformed cells trMCF and the cell lines which have undergone EMT. The number below the band shows the quantification by densitometry analysis. (F) Flow cytometric analysis of CD44/CD49f expression. The upper panel shows the isotype control, the lower panel shows the double staining of CD44 and CD49f. The experiment was repeated twice, one representative experiment was shown.

We then assessed the expression of CSC markers in xenografts and lung metastases. Consistent with what we have already reported, CD24 expression was low in the xenografts and lung metastases from XtMCF cells (Fig. 6A). For LmMCF cell model, there was a moderate expression of CD24 in xenografts, whereas a low expression in lung metastases was observed. CD44 was positive in both xenografts and lung metastases of XtMCF and LmMCF cells. EpCAM(VU1D9) was negative, whereas EpCAM{Abbiotec} and EpCAM(E144) were positive in xenografts and metastases from both cell lines. Of note, CD44, EGF‐like‐domain‐cleaved‐off EpCAM, and vimentin were stronger in the xenografts of LmMCF than in XtMCF. The same expression pattern of EpCAM was also evaluated and confirmed in MDA‐MB‐231 xenografts (Fig. 6B). In human primary TNBC tissues, the reactivity of EpCAM(VU1D9) in tumor cells which have lost E‐cadherin or have undergone EMT was significantly lower than that in tumor cells which show E‐cadherin expression (Fig. 6B).

It was reported that nuclear localization of EpCAM is implicated in the activation of Wnt signaling 22. We checked the beta‐catenin by immunofluorescence and showed membranous beta‐catenin was reduced in trMCF cells compared to MCF10F cells, and a strong staining in cytoplasm and nuclei was observed in mesenchymal‐like cells (Fig. 6C). Quantification of beta‐catenin fluorescent intensity showed it was significantly higher in the mesenchymal‐like cells than in MCF10F cells, and was higher in LmMCF than in bsMCF‐luc or XtMCF cells (Fig. 6D). The immunoblotting revealed a significant increase of Wnt signaling target gene Tcf4 (Fig. 6E) in transformed cells and cells underwent EMT, suggesting the activation of Wnt signaling during the progression.

### XtMCF and LmMCF cells are enriched for CD44+/CD49f+ cells

CD49f and CD133 are other two important CSC markers for basal‐like breast cancers 23. The CD44^POS^ CD49f^hi^CD133/2^hi^ cells isolated from ER‐negative patient tumors and xenografts are enriched for xenograft‐initiating cell capable of giving rise to TNBC xenografts 24. We evaluated the expression of CD44, CD49f, and CD133/2 by flow cytometric analysis. Consistent with the IF staining results, the rate of CD44‐positive cells was 55.3 ± 3.1% for MCF10F cells; it went up to 67.5 ± 1.3% for trMCF, 90.5 ± 3.2% for bsMCF, 89.5 ± 0.6% for bsMCF‐luc, 88.3 ± 1.5% for XtMCF, and 91.0 ± 1.1% for LmMCF. MDA‐MB‐231 and MDA‐MB‐468 had a faction of CD44‐positive cells (85.9 ± 1.2% and 92.5 ± 0.7% respectively) similar to our four TNBC cell lines, whereas T47D only had a small fraction of cells positive for CD44 (12.9 ± 0.8%). By contrast, over 90% of cells in the cell lines mentioned above were positive for CD49f, and there was no significant difference among these cell lines. CD133/2 was detected in 64.5 ± 0.9% of MDA‐MB‐468 cells but not in other cell lines. CD44^pos^CD49^hi^ cells from ER‐negative tumors have been shown to be tumorigenic 24; thus, we performed the double staining of CD44 and CD49f, and the results showed the fraction of CD44^+^/CD49f^+^ cells was low in luminal cell line T47D, in contrast to that it was high in basal cell lines. Interestingly, this fraction increased when cells were transformed and the EMT process was completed in our cell model (Fig. 6F); no difference was observed between the parental cell line bsMCF‐luc and two new cell lines XtMCF and LmMCF. Moreover, the fraction of CD44^+^/CD49f^+^ cells was very similar when comparing XtMCF and LmMCF with MDA‐MB‐231 and MDA‐MB‐468 cells. The results suggest CD44^+^/CD49f^+^ may be a good marker for both basal mammary stem cells and basal tumor‐initiating cells as MCF10F and trMCF present a significant fraction of double‐positive cells whereas both of them are not tumorigenic.

## Discussion

Triple‐negative breast cancer (TNBC) represents a heterogeneous group of cancers characterized by a lack of ER, PgR, and HER2 expression. Cluster analysis of human TNBC identified six subtypes displaying unique gene expression and ontologies 6. Approximately 80% of TNBC show features of basal‐like cancers 25. Transcriptional profile analysis assigned twenty‐one TNBC cell lines into three clusters: luminal, basal A, and basal B 16, 26, 27. Basal A contains cell lines such as BT‐20, Sum149, and MDA‐MB‐468, which preferentially expresses genes such as *CK5/6*,* CK14*, and *EGFR*. Basal B includes cell lines such as MDA‐MB‐231, Sum159pt, and Hs578t, which preferentially expresses genes such as *CD44*,* VIM*, and *SNAI2*, and exhibits a stem cell‐like profile 26. This classification of TNBC cell lines is closely associated with cell morphology and invasive potential. Basal B cells have a more mesenchymal‐like appearance and are less differentiated and much more invasive compared to the other two clusters. Analysis of the relationship between TNBC cell lines and tumor subtypes showed most of basal A and basal B cell lines resemble basal‐like tumors 16, indicating that TNBC cell lines are suitable for investigations of subtype‐specific cancer cell biology.

Although there are over twenty commercially available TNBC cell lines, MDA‐MB‐231 is the most widely used in vitro and in vivo. In BALB/CAJCI‐nu/nu mice, it took five weeks to form a xenograft around 6.5 mm in diameter with the subcutaneous injection of 5 × 10^6^ MDA‐MB‐231 cells 28. MDA‐MB‐468 cells had a growth speed similar to MDA‐MB‐231 in the same mouse strain 28. The growth speed of MDA‐MB‐231 xenograft in CB17/SCID was almost the same as in nude mice, while BT‐549 cells grew a little bit slower than MDA‐MB‐231 cells in CB17/SCID mice 29. Sum149 and Sum159 are two highly tumorigenic cell lines, it was reported the injection of 1 × 10^5^ cells in nonobese diabetic SCID mice could produce tumors in 3/4 and 5/6 mice, respectively 30. But these two cell lines are mainly used for the study of inflammatory breast cancer 9, 31.

Considering the heterogeneity and complexity of TNBC, more cell lines and animal models are needed. We herein report the establishment of a progressive TNBC model consisting of normal MCF10F, transformed cell line trMCF, and tumorigenic cell lines bsMCF, XtMCF and LmMCF. Compared to the nine tumorigenic TNBC cell lines mentioned in the introduction and in this discussion, XtMCF and LmMCF cell lines are the most tumorigenic and metastatic. The expression of CK18 confirmed the epithelial origin of this cell model. We observed that CK18 was downregulated in bsMCF cell line and its derivatives. Furthermore, CK18 was lost in the lung metastases, whereas still present in the xenografts of both XtMCF and LmMCF cells, suggesting downregulation of CK18 may be related to breast tumor progression. This idea is supported by Woelfle's study, which found that downregulation of CK18 was significantly correlated to advanced tumor stage and high grade 32. Of interest, CK5 was progressively downregulated in our cell model. Our previous study showed CK5‐positive cell number was inversely correlated to clinical stage of TNBC 33. Aguiar et al. also found CK5/6 expression was negatively associated with the probability of invasion 34, suggesting that our cell model reflects features of TNBC progression.

The EMT process is not only closely related to cancer invasion and metastasis but also conferred to the generation of CSC 20, 35, 36. As bsMCF‐luc, XtMCF, and LmMCF have undergone EMT, we evaluated their CSC properties and the results showed that they could form tumorspheres, and the number of tumorspheres was progressively increasing from bsMCF‐luc to XtMCF and LmMCF cells, consistent with in vivo tumorigenic and metastatic potential. The colony formation assay showed fewer colonies were produced by XtMCF and LmMCF cells compared to bsMCF‐luc, indicating the number of colonies in methylcellulose may not be always associated with tumorigenicity. The growth pattern of colonies should also be considered; for example, budding from the surface of colonies was frequently observed in XtMCF and LmMCF colonies, which may indicate its aggressiveness and tumorigenicity.

We postulated that the evaluation of CSC markers would give us a rationale for the high tumorigenic and metastatic potential of these two cell lines. Our results showed that the bsMCF‐luc and XtMCF cells were CD24^low^/CD44^+^, whereas LmMCF cells were CD44^+^ with moderate CD24 expression. CD24^‐/low^/CD44^+^ has been frequently used as CSC markers of breast cancers 37, 38, 39. However, it was shown that the percentage of CD24^‐/low^/CD44^+^ associates with a basal‐like phenotype, not tumorigenicity, but CD24^‐/low^/CD44^+^/EpCAM^+^ cells enrich for tumorigenicity 30, 37. Lin et al. 40 showed EpCAM induces expressions of reprogramming factors and EMT genes, regulates EMT progression and tumorigenesis. In addition, EpCAM can be cleaved at several sites, and the nuclear translocation of cytoplasmic domain (EpCID) associates with Wnt pathway and promotes cell proliferation and tumor formation in mice 22, 41. One of EpCAM cleavage sites between two arginine residues (AA80 and AA81) was detected and described in the late 1980s, but the functional consequence is still unknown 42. Interestingly, we observed the expression of EpCAM in the cell lines we examined by immunofluorescence staining and WB, but the EGF‐like domain of EpCAM was absent in mesenchymal‐like cells, suggesting the EGF‐like domain might be cleaved off from the cleavage site between AA80 and AA81. This was supported by Keller and collaborators, who showed that mesenchymal‐like cells do not express EpCAM using antibody EpCAM(VU1D9) 43. Gorges et al. 44 also showed that circulating tumor cells escape from EpCAM‐based detection due to EMT. The issue that circulating tumor cells are undetectable in some patients with metastatic breast cancer using CellSearch assay 45 and the fact that CellSearch test does not recognize normal‐like breast tumor cells (MDA‐MB‐231) 46 have been noticed and made demand on developing new approaches to detect circulating cancer cell 47, 48, 49, 50. The majority of commercial antibodies for EpCAM react with overlapped or partly overlapped epitope at EGF‐like domain 21. This may result in failing detection of EpCAM in cells which have undergone EMT. Our study indicates that the EGF‐like‐domain‐cleaved‐off EpCAM may be associated with the EMT process. Furthermore, although the total level of EpCAM is low in mesenchymal‐like cells, the subcellular localization of EpCAM may be more important to the EpCAM nuclear signaling as strong activation of Wnt signaling was observed in these cells.

Meyer et al. 24 reported the CD44^POS^CD49f^hi^CD133/2^hi^ population obtained from two pleural effusions and four xenografts of ER‐negative breast cancers consistently enriched for xenograft‐initiating cells. However, there was marked difference in the tumorigenicity across the xenografts. We did not observe the expression of CD133 in our cell model and MDA‐MB‐231 cell line, consistent with Croker's study that found that MDA‐MB‐231 did not demonstrate expression of CD133 51. It was reported in Liu's study that the rate of CD133^+^ population was 25.4% in MDA‐MB‐231 cells, and it varied among the MDA‐MB‐231 clones with different morphology 52. The discrepancy regarding the expression of CD133 in MDA‐MB‐231 cells may be partly due to the sources of MDA‐MB‐231 cells and CD133 antibody used in different laboratories. MDA‐MB‐468 cells were shown consistently expressing CD133/2 relatively higher than other cell lines 51, 53. The observation that CD44^+^/CD49f^+^ population differentiated significantly between basal and luminal cell lines indicates that CD44^+^/CD49f^+^ may be one of the important properties of basal stem cells. It is interesting that this population is preserved during the transformation in our TNBC model, suggesting it might be the fact that when normal mammary stem cells transform to cancer stem cells, the stem cell property is preserved, besides that, the transformed stem cells gain the phenotype of malignancy. Atkinson et al. also reported that CD44^+^/CD49f^+^CD133/2^+^ were present in the normal adjacent tissues in patients with TNBC to a much greater extent than in the ER+ samples, supporting the hypothesis that stem cells present in normal breast tissue may be either the cell of origin of cancer or promote the development of breast cancer 54.

Another interesting observation was the nuclear E‐cadherin staining in xenografts of LmMCF and MDA‐MB‐231 cell lines, which was consistent with the finding that nuclear translocation of cleaved cytoplasmic domain of E‐cadherin plays oncogenic roles 55.

Taken together, we present the development and characterization of two highly tumorigenic and metastatic basal B TNBC cell lines, XtMCF and LmMCF. To the best of our knowledge, they are the most tumorigenic and metastatic TNBC cell lines compared to all reported cell models used for TNBC studies. In addition, the normal and early‐stage counterparts of these two cell lines are also available. Altogether, these cell lines can be used to study the evolution of TNBC, investigate molecular pathways at different stages of transformation and progression in a relatively constant genetic background, and most importantly, identify new treatments for TNBC. In addition, XtMCF and LmMCF cell lines present CSC properties and can be used for developing CSC targeted therapy. The finding that the EGF‐like domain of EpCAM is cleaved off in cancer cells which have undergone EMT also provides new insights in research of EMT and CSC, two important fields in cancer biology.

## Conflict of Interest

The authors declare no potential conflict of interests.

## Supporting information


**Appendix S1**. Cell culture
**Table S1**. Antibodies information
