# Supplementary material for: Development and characterization of two human triple‐negative breast cancer cell lines with highly tumorigenic and metastatic capabilities
Source: Cancer Med. 2016 Jan 18;5(3):558–73. doi: 10.1002/cam4.616 (PMC4799943; doi:10.1002/cam4.616)
Supplement: Supplementary file 1 — Appendix S1. Cell culture Table S1. Antibodies information [file CAM4-5-558-s001.doc]

**Supplementary methods**

**Cell culture**

MCF-10F, trMCF, and bsMCF cells were maintained in DMEM:F12 media (1:1 Gibco/BRL, Gaithersburg, MD) supplemented with 5% horse serum (Gibco), 100 ng/ml cholera toxin (ICN Biomedicals, Cleveland, OH), 10µg/ml insulin (Sigma, St. Luis, MO), 0.5 μg/ml hydrocortisone (Sigma), 20 ng/ml epidermal growth factor (Gibco), 1.05mM CaCl_2_, and antibiotics (penicillin, 100 U/ml; streptomycin, 100 µg/ml; amphotericin, 0.25 µg/ml; Sigma). bsMCF cells were transfected with pGL4.51(luc2/CMV/Neo) vector (Promega, San Luis Obispo, CA) and maintained in media with 800 µg/ml G418, named bsMCF-luc cells. T47D, HCC1954, MDA-MB-231, MDA-MB-468, and Hs578t were maintained in RPMI-1640 with 10% FBS. MCF7 was maintained in DMEM with 10% FBS. SK-BR-3 was cultured in McCoy’s 5a media with 10% FBS. Sum149pt and Sum159pt were cultured in Ham’s F12 media with 10% FBS, 1 µg/ml hydrocortisone, 50 µg/ml insulin. All Cells were maintained in a 37ºC incubator supplied with 5% CO_2_. MCF10F, T47D, MCF7, SK-BR-3, MDA-MB-231, MDA-MB-468, and Hs578t were obtained from ATCC. HCC1954 was from American Type Culture Collection (ATCC). Sum149pt and Sum159pt were obtained from Asterand (Detroit, MI).

**Colony forming efficiency in methylcellulose**

Single cells were suspended in 0.8% methylcellulose dissolved in DMEM/F12 media, plated at 1500 cells/well in four replicates onto 24-well plates pre-coated with 5% agar. 1 ml of media was added to each well after methylcellulose solidified. Media was refreshed every three days. The colony formation was examined under a microscope. At the end of examination period, cells were stained with neutral red solution; images were acquired using NIKON ECLIPSE TS100 microscope. The number and size of colonies were measured using MetaMorph.

**Immunofluorescence (IF) staining**

Cells were plated on chamber slides. After 3 days of culture, cells were fixed with 10% buffered formalin, permeabilized with 0.25% Triton X-100, blocked with 5% goat serum for 30 minutes, and stained with antibodies of interest at 4°C overnight. The expression of proteins was detected using Alex Fluor® 488 goat anti-mouse antibody or Alex Fluor® 555 goat anti-rabbit antibody. Nuclei were counterstained with 4',6-diamidino-2-phenylindole (DAPI). Fluorescent images were captured using Olympus BX53 fluorescent microscope with monochrome camera (Olympus Corporation) and MetaMorph Software version 7.7.8.0 (Molecular Devices, Sunnyvale CA). The fluorescent intensity was quantified using MetaMorph.

Supplementary Table 1, Antibodies information

|  | Antibody name(clone) | Provider | Catalog# | Dilution (IF) | Dilution  (WB) | Dilution  (IHC) |
| --- | --- | --- | --- | --- | --- | --- |
| 1 | E-cadherin(36) | BD Biosciences | 610182 | 1:200 | 1:1000 |  |
| 2 | E-cadherin(36) | BioGenex | AM390-10M |  |  | R-T-U |
| 3 | vimentin(V9) | DAKO | M0725 | 1:200 | 1:1000 |  |
| 4 | vimentin(V9) | BioGenex | AM074-5M |  |  | R-T-U |
| 5 | ER alpha(EPR703(2)) | BioGenex | AN509-5M | R-T-U |  | R-T-U |
| 6 | PgR(PgR636) | Biocare | PM343 | R-T-U |  | R-T-U |
| 7 | HER2(EP1045Y) | BioGenex | AN471-5N | R-T-U |  | R-T-U |
| 8 | CK5(EPR1600Y) | BioGenex | AN584-5N | R-T-U |  | R-T-U |
| 9 | CK18(EPR1626) | abcam | ab133263 | 1:400 | 1:1000 | 1:250 |
| 10 | CK19(RCK108) | BioGenex | AN484-10M | R-T-U |  | R-T-U |
| 11 | CD24 | Abbiotech | 251181 | 1:200 |  | 1:150 |
| 12 | CD44(DF1485) | BioGenex | AM310-5M | R-T-U |  | R-T-U |
| 13 | EpCAM(VU1D9) | Cell Signaling | 2929 | 1:400 | 1:1000 | 1:200 |
| 14 | EpCAM{Abbiotech} | Abbiotech | 251617 | 1:200 |  | 1:200 |
| 15 | EpCAM(E144) | abcam | ab32392 | 1:200 | 1:400 |  |
| 16 | EpCAM(E144) | BioGenex | AN489-5M |  |  | R-T-U |
| 17 | Beta-catenin(CAT-5H10) | Life technologies | 13-8400 | 1:200 |  |  |
| 18 | Tcf4(EP2033Y) | EMD Millipore | 04-1080 |  | 1:1000 |  |
